# Identifying perianal fistula complications in pediatric patients with Crohn’s disease using administrative claims

**DOI:** 10.1371/journal.pone.0219893

**Published:** 2019-08-14

**Authors:** Jeremy Adler, Hannah K. Jary, Sally J. Eder, Shiming Dong, Emily Brandt, Jessica K. Haraga, Kevin J. Dombkowski

**Affiliations:** 1 Department of Pediatrics and Communicable Diseases, Division of Pediatric Gastroenterology, University of Michigan, Ann Arbor, Michigan, United States of America; 2 Susan B. Meister Child Health Evaluation and Research (CHEAR) Center, University of Michigan, Ann Arbor Michigan, United States of America; Osaka Medical Center for Cancer and Cardiovascular Diseases, JAPAN

## Abstract

**Background:**

Although perianal fistulas occur commonly in pediatric Crohn’s disease (CD), evaluations of health services have been limited since no validated claims-based methods exist for identifying cases.

**Objective:**

To develop and validate accurate case definitions for perianal fistulas among pediatric patients with CD from administrative claims.

**Methods:**

Retrospective cohort study in which we developed and tested candidate case definitions for perianal fistula. Patients (age 5–21 years between 2005–2012) with CD enrolled in Michigan Medicaid with healthcare at University of Michigan were identified via claims. Medical records were obtained from all identified patients, whose entire records were abstracted. Medical record evidence for perianal fistula was considered the “gold standard” against which candidate case definitions were compared. The reference case definition of perianal fistula (ICD9 565.1) and candidate case definitions were evaluated.

**Results:**

Of 843 patients identified via claims, 274 (33%) met CD criteria for inclusion. The true perianal fistula rate among CD patients was 18% (n = 49). The top-performing candidate case definition identified 15% (n = 42), had sensitivity of 77.6%, specificity of 98.2%, positive predictive value (PPV) 90.5%, negative predictive value (NPV) 95.3%, and area under receiver operator characteristic curve (ROC) of 0.88. In contrast, the reference case definition identified 9% (n = 26), sensitivity 51.0%, specificity 99.6%, PPV 96.2%, NPV 90.3%, and had an area under ROC of 0.75.

**Conclusions:**

We demonstrated that it is feasible to use administrative claims data to accurately identify pediatric patients with perianal fistula complications. Claims-based case definitions were found to be highly accurate through medical record review, providing a high degree of confidence for future studies where chart review is not feasible. These claims-based methods can be applied to claims data in other settings for the evaluation of health services utilization as well as to assess the comparative effectiveness of prevention and treatment strategies.

## Introduction

Gastrointestinal fistulas are abnormal connections between the bowel and adjacent structures such as the skin, bladder, vagina, fallopian tubes, or to other loops of bowel, and have significant negative impact on their quality of life.[1–3] Fistulas are a major cause of morbidity among patients with Crohn’s disease, a type of inflammatory bowel disease (IBD). Fistulas due to Crohn’s disease most commonly develop in the perianal region, causing severe infections, fecal incontinence, perianal discharge, negative self-image and social isolation; they can lead to the need for numerous surgeries as well as infertility.[4, 5] Perianal fistulas often cause serious, debilitating complications that are difficult to treat, leading to increased costs.[6, 7]

Despite the profound effects on quality of life and health care costs, very little is known about the epidemiology of fistulas due to Crohn’s disease. Prospective adult studies found that perianal fistulas commonly develop among 15% within 5 years and 23% within 10 years after Crohn’s disease diagnosis.[1, 8] Onset of Crohn’s disease in childhood is associated with more aggressive perianal fistula development, with fistulas occurring in as many as 20–31% of children within 5–7 years after Crohn’s disease diagnosis.[9–12] Population studies of perianal fistulas are lacking.[13] Based on national estimates of pediatric Crohn’s disease prevalence, this suggests that there are more than 10,000 children with perianal fistulas due to Crohn’s disease in the United States.[14]

While these studies provide some important initial insights, population-based methods to assess the prevalence of fistulizing Crohn’s disease among children and related outcomes do not exist. The few population-based studies of perianal fistulas that have been conducted in claims-based data were not validated with medical record review and therefore their accuracy is unknown.[6, 15, 16] Consequently, a validated claims-based approach to identify perianal fistulizing Crohn’s disease cases is not currently available for evaluation of health service utilization or to target interventions among children. With that in mind, the objective of this study was to develop and validate a claims-based method for identifying perianal fistulas among pediatric patients with Crohn’s disease.

## Materials and methods

We developed and tested alternative claims-based methods for identifying perianal fistula complications of pediatric patients with Crohn’s disease among a statewide population of children enrolled in Michigan Medicaid. We validated our claims-based methods among the sub-population of pediatric patients with Crohn’s disease at the University of Michigan who were enrolled in the Michigan Medicaid program during the 2005–2012 study period. This study was approved by the institutional review board (IRB) at the University of Michigan (HUM00079109) and the Michigan Department of Health and Human Services (MDHHS). The IRBs granted a waiver of consent in order allow the linkage of claims data to the electronic medical records and to permit review of the medical records necessary for comparison with claims data. While permission was granted to allow medical record validation, these data cannot be made publicly available due to patient privacy considerations. All aggregate data used in this study are reported in the manuscript, tables and figures.

### Case definition development

Previous claims-based studies of perianal fistula in Crohn’s disease have previously used a single diagnosis code (ICD-9 diagnosis = 565.1) to identify fistula cases, although the accuracy of this method is unknown.[6, 15] For the purposes of this study, we refer to this as the “reference case definition” against which all of our candidate case definitions were compared. We developed candidate case definitions for perianal fistulas to explore the relative accuracy of each for identifying cases using administrative claims data.

Our candidate case definitions were based upon a review of the medical literature in which we summarized the range of approaches to evaluation and treatment of perianal fistulas among patients with Crohn’s disease. These approaches ultimately served as the basis for identification of cases based upon the procedure and medication codes reported in claims data. In total, our review yielded 99 candidate strategies for which we identified the relevant diagnosis, procedure and medication codes that could be used with administrative claims data to identify perianal fistula cases (Table 1). Detailed descriptions of relevant codes and case definitions are provided in supplemental tables (S1 and S2 Tables).

**Table 1:** Case definitions for perianal fistulas, categorized by claims requirements.

Definition Category	Case Definitions	Medications^b^	Perianal fistula^c^	Perianal lesions^d^	Genital fistula^e^	Procedures^f^	Imaging^g^
A	Reference case definition^a^		●				
B	Perianal fistula (composite)		●	●	●		
C	Medication only (no perianal lesion)	●					
D	Perianal fistula AND medication	●	●		●		
E	Perianal fistula/lesion AND medication	●	●	●	●		
F	Procedure					●	
G	Procedure AND perianal lesion			●		●	
H	Procedure AND perianal fistula		●		●	●	
I	Procedure AND perianal fistula/lesion		●	●	●	●	
J	Procedure OR perianal fistula/lesion		●	●	●	●	
K	Imaging						●
L	Imaging AND perianal lesion			●			●
M	Imaging AND perianal fistula		●		●		●
N	Imaging AND Perianal fistula/lesion		●	●	●		●
O	Imaging AND medication	●					●
P	Imaging AND perianal lesion AND medication	●		●			●
Q	Imaging AND perianal fistula AND medication	●	●		●		●
R	Imaging AND perianal fistula/lesion AND medication	●	●	●	●		●
S	Other combinations	●	●	●	●	●	●

● represents required category of claims in order to meet given case definition.

^a^Does not include perirectal abscess.

^b^Medications include Immunomodulator, anti-TNFα, antibiotics.

^c^Perianal fistula includes perirectal abscess.

^d^Perianal lesions include skin tags, hemorrhoids.

^e^Genital fistula includes genital abscess.

^f^Procedures include fistulotomy, fistulectomy, lesion removal, incision and drainage, abscess.

^g^Imaging includes CT, MRI, fluoroscopy.

Anti-TNFα, anti-tumor necrosis factor alpha; CT, computed tomography imaging; MRI, magnetic resonance imaging

#### Evaluation strategies

We considered a broad range of evaluation strategies to identify potential cases of perianal fistulas. Patients with Crohn’s disease who have suspected or identified perianal fistulas and/or abscesses are most commonly evaluated by either cross sectional imaging (computed tomography [CT] or magnetic resonance imaging [MRI]) or endoscopic ultrasound (EUS).[17–23] Historically fluoroscopic fistula studies have been performed such as fistulography or barium enema.[17] Since the frequency with which these are still performed is unknown, we included this as a potential diagnostic technique.

#### Treatment strategies

We also considered a diverse array of potential treatment options in our approach to identify potential perianal fistula cases. The literature supports treating patients with perianal fistula with aggressive medical therapy for Crohn’s disease including immunomodulator and/or anti-tumor necrosis alpha (TNFα) therapy.[20, 24–26] There is also evidence to support the use of antibiotics including metronidazole and/or ciprofloxacin.[26] Various surgical techniques have been used to treat perianal fistulas and abscesses including incision and drainage of an abscess, fistulotomy, fistulectomy, seton placement, advancement flaps, and injection of fibrin glue.[17, 27] We also included diagnoses of and procedures for treatment of other perianal lesions including skin tags and hemorrhoids, since perianal Crohn’s disease complications can be mistaken for these lesions.[28–30]

### Study population

We acquired all Michigan Medicaid administrative claims for pediatric patients 5–21 years with Crohn’s disease between January 1, 2005 and December 31, 2012. As an initial screening, potentially eligible patients were required to have at least three encounters with diagnosis of IBD. This method has previously been validated by medical record review with a high degree of sensitivity, specificity, and negative predictive value (NPV), though it has a low positive predictive value (PPV).[14, 31, 32] The high NPV of this definition makes it very unlikely that we missed cases of Crohn’s disease. The low PPV makes this definition insufficient to be confident that there were no false positives, and all cases identified truly had Crohn’s disease. To address this, we then identified these patients in the University of Michigan Health System and reviewed their electronic medical records to verify whether or not they had Crohn’s disease during the study period. Through this two-step process of identifying patients, we had a high degree of confidence regarding patients’ Crohn’s disease status.

### Classification of Claims

We used Medicaid claims to classify perianal fistula cases based upon codes indicating the evaluation and/or treatment of Crohn’s disease. Our general approach to claims classification built upon our prior experiences with classifying chronic disease cases using administrative claims.[33–38] All claims for Crohn’s disease patients during the study period were acquired, irrespective of diagnosis, procedure or medication. Medicaid claims for all medical care, both within and outside of the University of Michigan Health System, were evaluated. Information on perianal fistula outcomes was collected irrespective of diagnosis, procedure or medication. Claims for each patient were classified according to the candidate case definitions as detailed in supplemental table (S3 Table).

### Medical record abstraction

We reviewed the entire medical record during the study timeframe for each patient, including all inpatient and outpatient clinician notes. Diagnostic information was sought from descriptions of physical examination, recorded diagnoses, endoscopy reports, imaging reports, operative reports, etc. The diagnosis of Crohn’s disease was confirmed according to the Porto criteria, guidelines for diagnosis that include endoscopic evaluation with biopsy in addition to evaluation of the small bowel with medical imaging.[39] Crohn’s disease phenotype was categorized according to Paris classification.[40] Explicit evidence of any perianal complication of Crohn’s disease within the study period was sought from the entire medical record. The presence or absence of perianal fistula in the medical record was considered the “gold standard” against which all candidate claims-based case definitions were to be compared.

During medical record review, a patient was considered to have a perianal fistula if either 1) a perianal fistula and/or 2) a perianal abscess was documented. For the purposes of this study, a perianal abscess without explicit identification of a fistula tract was still considered as having a perianal fistula.[17, 39] Pilonidal abscess or other skin abscesses distant from the anus were not considered to be a perianal fistulas unless a fistula tract was explicitly identified. However genital fistulas and/or abscesses were considered perianal lesions if they were documented to be due to Crohn’s disease in the medical record.[19, 41, 42] Documentation of a patient’s self-report of past history of fistula was not sufficient to be considered as true fistula unless documented evidence of perianal fistula or abscess was found in the medical record.

### Evaluation of candidate definitions

Our candidate claims-based case definitions for perianal fistulizing Crohn’s disease were evaluated using our sample of medical records as the gold standard. Though the medical record was abstracted for the entire study timeframe, the analyses were limited to periods of Medicaid enrollment. Perianal fistulas documented outside of the time period of Medicaid enrollment were not included as outcomes. Following an approach successfully employed in a prior study, measures of performance were calculated for each candidate case definition, including sensitivity, specificity, PPV, NPV, and area under the receiver operating characteristic (ROC) curve.[38]

## Results

### Study population

**Fig 1:**
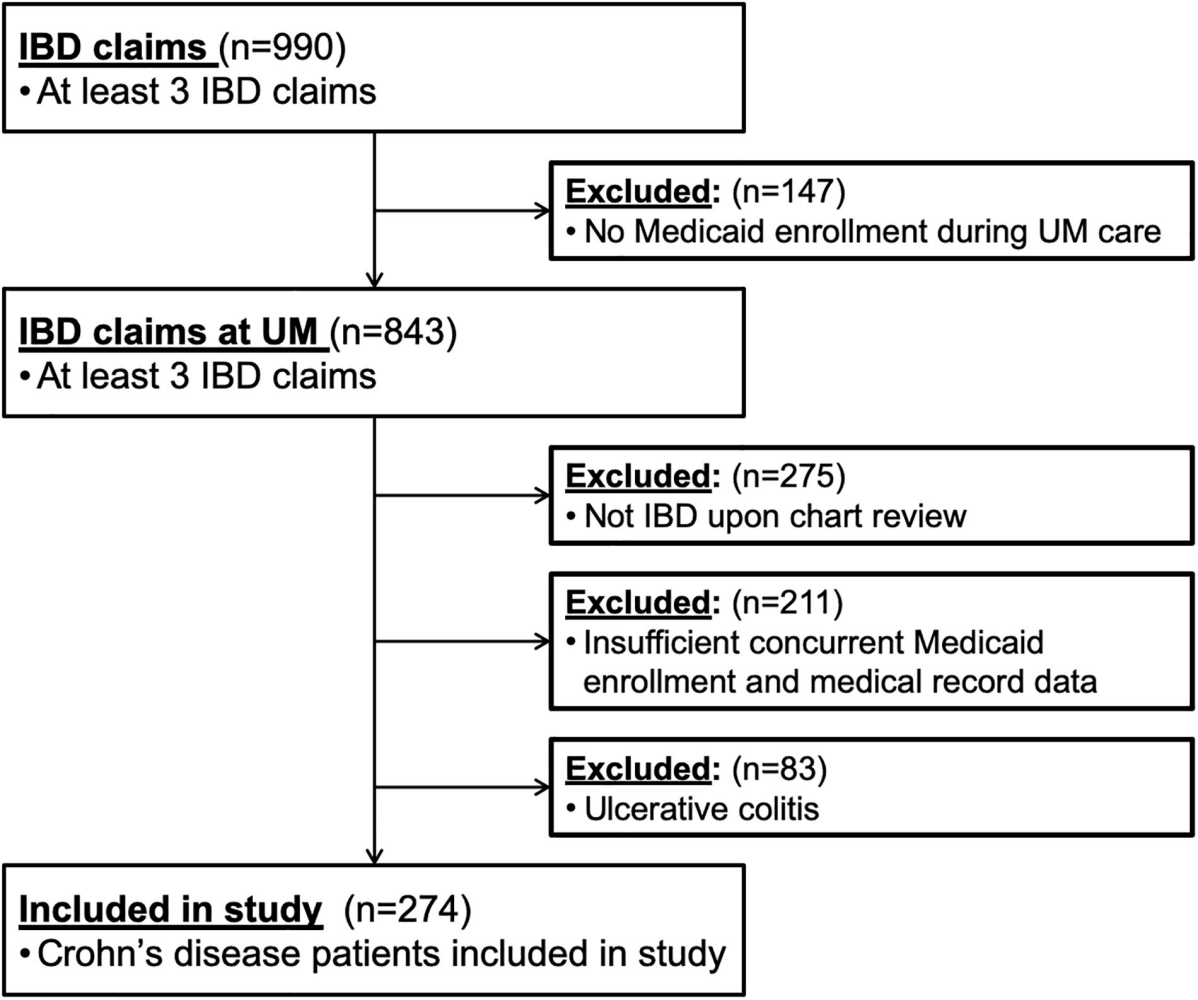
Study population (2005–2012).

A total of 843 patients at the University of Michigan were identified in Michigan Medicaid claims as having at least 3 claims for IBD during the study period (Fig 1), all of whose medical records were abstracted. Among those cases 274 (33%) had confirmed Crohn’s disease diagnosis and adequate concurrent time periods of Medicaid enrollment for inclusion in the study. Of these included patients, the mean age at study enrollment was 15.6 ±4.1 years; 132 (48%) were female, 187 (68%) were white, 47 (17%) were black, 3 (1%) were Asian, and 37 (14%) had missing or unknown data for race (Table 2). The characteristics of the patient population contrasting confirmatory evidence determined through claims versus medical records is summarized in Table 3.

**Table 2:** Characteristics of the patient population based on claims and Medical record evidence.

	Claims Evidence	Medical Record Evidence
**Perianal Lesion Evidence**		
Perianal fistula^a^ (N = 274)	26 (9%)	39 (14%)
Perirectal abscess (N = 274)	32 (12%)	44 (16%)
Perianal fistula OR abscess (N = 274)	32 (12%)	49 (18%)
Perianal lesion^a^^b^ (N = 274)	16 (6%)	102 (37%)
Perianal lesion OR abscess^b^ (N = 274)	34 (12%)	89 (32%)
Perianal lesion OR abscess OR fistula (N = 274)	36 (13%)	102 (37%)
**Medication Evidence**		
Anti-TNFα (N = 274)	156 (57%)	135 (49%)
Antibiotic (N = 166)	101 (61%)	82 (51%)
Immunomodulator (N = 166)	106 (64%)	93 (56%)
Immunomodulator AND antibiotic (N = 166)	75 (45%)	51 (30%)
Immunomodulator AND anti-TNFα (N = 166)	69 (42%)	63 (38%)
Immunomodulator OR anti-TNFα (N = 166)	136 (82%)	117 (70%)
Anti-TNFα AND antibiotic (N = 166)	64 (39%)	77 (28%)
Anti-TNFα OR antibiotic (N = 166)	136 (82%)	187 (68%)
(Immunomodulator OR anti-TNFα) AND antibiotic (N = 166)	84 (51%)	78 (28%)
**Imaging Evidence**		
Computed tomography imaging (CT) (N = 274)	120 (44%)	107 (39%)
Magnetic resonance imaging (MRI) (N = 274)	106 (39%)	104 (38%)
Fluoroscopy (N = 274)	22 (8%)	30 (11%)
**Procedure Evidence**		
Seton (N = 274)	10 (4%)	7 (3%)
Excision of lesion (N = 274)	3 (1%)	6 (2%)
Fistula procedure (N = 274)	7 (3%)	12 (4%)
Hemorrhoidectomy (N = 274)	2 (1%)	1 (<1%)
Excision lesion OR fistula procedure (N = 274)	11 (4%)	14 (5%)
Excision lesion OR fistula procedure OR seton placement (N = 274)	15 (5%)	19 (7%)

S.D., standard deviation; Anti-TNFα, anti-tumor necrosis factor alpha;

^a^Excludes abscess;

^b^Excludes fistula; N, number

**Table 3:** Patient characteristics in electronic medical record.

		No Perianal Fistula (N = 225)	Perianal Fistula (N = 49)	p-value
Age, years ± SD		15.8 ± 4.1	14.7 ± 3.9	0.09
Female gender, N (%)		105 (46.7%)	27 (55.1%)	0.28
Race, N (%)	Black	35 (74.5%)	12 (25.5%)	0.039
	White	152 (81.3%)	35 (18.7%)	
	Other/unknown	38 (95.0%)	2 (5.0%)	
Medicaid enrollment, years ± SD		6.1 ± 2.6	7.1 ± 2.2	0.007
Phenotype*, N (%)	Nonstricturing, nonpenetrating (B1)	154 (88.5%)	20 (11.5%)	0.001
	Stricturing (B2)	44 (75.9%)	14 (24.1%)	
	Internal penetrating (B3)	9 (64.3%)	5 (35.7%)	
	Penetrating and stricturing (B2B3)	18 (64.3%)	10 (35.7%)	
Medication use, N (%)	Immunomodulator	73 (54.9%)	20 (60.6%)	0.55
	Anti-TNFα	94 (41.8%)	41 (83.7%)	<0.001
	Antibiotic	50 (39.1%)	32 (97.0%)	<0.001

*Phenotype categorized according to Paris classification; N, number; SD, standard deviation; Anti-TNFα, anti-tumor necrosis factor alpha

### Claims findings

Among the 274 patients with claims reporting Crohn’s disease, 26 (9%) had at least one claim for a perianal fistula, and 32 (12%) had claims for perirectal abscess. Lesions other than fistula or abscess, such as a perianal skin tag, or hemorrhoid were reported among 16 patients (6%). A subset of patients (39%) had missing data for immunomodulators and antibiotics. Among those with complete medication data (N = 166), 108 (64%) with at least one claim for an immunomodulator, and 101 (61%) for an antibiotic. Among all 274 patients, 156 (57%) with at least one claim for an anti-TNFα medication. Overall, 10 patients (4%) had seton procedures, 7 (3%) had fistula closure procedures, 3 (1%) had excision of a perianal lesion and 2 (1%) had hemorrhoidectomy. Imaging procedures included 120 (44%) CT, 106 (39%) MRI, 22 (8%) fluoroscopic procedures, and no EUS procedures.

### Medical record validation

Using the medical record as the gold standard, the true fistula rate among Crohn’s disease patients identified through claims was 18% (N = 49). Among the 274 patients with Crohn’s disease, the reference case definition for perianal fistula (one ICD-9 diagnosis code = 565.1) identified 26 patients (9%) with perianal fistula, 1 of whom was determined to be false positives through medical record review. This reference case definition had a sensitivity of 51.0%, specificity 99.6%, PPV 96.2%, NPV 90.3%, and area under the ROC curve of 0.75.

**Fig 2:**
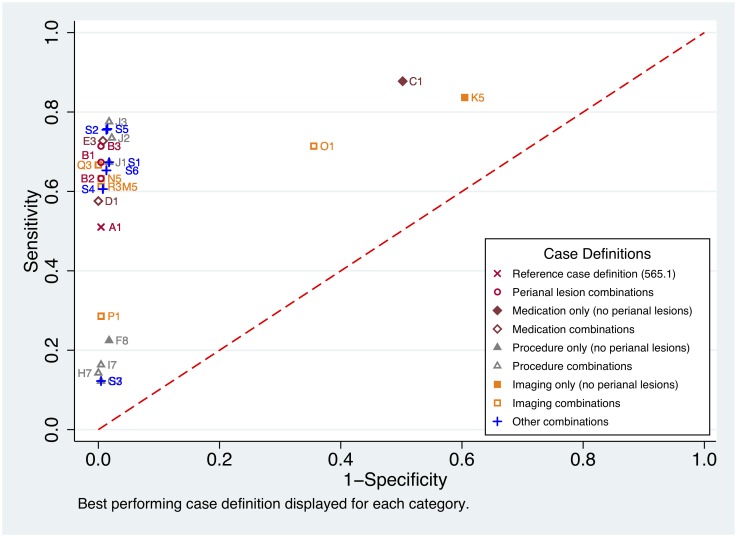
Receiver operating characteristic (ROC) curve for Crohn’s disease patients.

Measures of performance of the candidate definitions varied widely among the 99 candidate case definitions tested (S4 Table). Sensitivity ranged from 2.0% to 98.0%, specificity from 12.4% to 100.0%, PPV from 13.6% to 100.0%, NPV 81.2% to 96.7%, and area under the ROC curve from 0.48 to 0.88. The top case definitions, based on the measures of performance were definitions B1, B2, B3, J1, J2, J3, M5, N5, R5, and S1 through S6 (Table 4). The range of performances across case definitions within each case definition category is summarized in S4 Table and shown in the ROC curve (Fig 2).

**Table 4:** Measures of performance for top performing case definitions and reference case definition.

Cat	Description	Sensitivity (95% CI)	Specificity (95% CI)	PPV (95% CI)	NPV (95% CI)	Area under ROC Curve (95% CI)
A1	Reference case definition	51.0% (36.3–65.6)	99.6% (97.5–100.0)	96.2% (80.4–99.9)	90.3% (85.9–93.7)	0.75 (0.68–0.82)
B1	Perianal fistula OR perirectal abscess	67.3% (52.5–80.1)	99.6% (97.5–100.0)	97.1% (84.7–99.9)	93.3% (89.4–96.1)	0.83 (0.77–0.90)
B2	Perianal fistula OR perirectal abscess OR genital fistula OR genital abscess	63.3% (48.3–76.6)	99.6% (97.5–100.0)	96.9% (83.8–99.9)	92.6% (88.5–95.5)	0.81 (0.75–0.88)
B3	Perianal fistula OR perirectal abscess OR genital fistula OR genital abscess OR perianal lesion	71.4% (56.7–83.4)	99.6% (97.5–100.0)	97.2% (85.5–99.9)	94.1% (90.3–96.7)	0.85 (0.79–0.92)
J1	Seton OR fistula closure OR lesion excision OR perianal fistula/lesion	67.3% (52.5–80.1)	98.2% (95.5–99.5)	89.2% (74.6–97.0)	93.2% (89.3–96.1)	0.83 (0.76–0.89)
J2	Seton OR fistula closure OR lesion excision OR perianal fistula/abscess/lesion	73.5% (58.9–85.1)	97.8% (94.9–99.3)	87.8% (73.8–95.9)	94.4% (90.6–97.0)	0.86 (0.79–0.92)
J3	Seton OR fistula closure OR lesion excision OR perianal/ genital fistula/abscess/lesion	77.6% (63.4–88.2)	98.2% (95.5–99.5)	90.5% (77.4–97.3)	95.3% (91.7–97.6)	0.88 (0.82–0.94)
M5	(CT OR MRI) AND perianal fistula	61.2% (46.2–74.8)	99.6% (97.5–100.0)	96.8% (83.3–99.9)	92.2% (88.1–95.2)	0.80 (0.73–0.87)
N5	(CT OR MRI) AND perianal fistula/lesion	63.3% (48.3–76.6)	99.6% (97.5–100.0)	96.9% (83.8–99.9)	92.6% (88.5–95.5)	0.81 (0.75–0.88)
R3	(CT OR MRI) AND perianal fistula/lesion AND (anti-TNFα OR antibiotic)	61.2% (46.2–74.8)	99.6% (97.5–100.0)	96.8% (83.3–99.9)	92.2% (88.1–95.2)	0.80 (0.73–0.87)
S1	Seton OR fistula procedure OR fistula or abscess	67.3% (52.5–80.1)	98.2% (95.5–99.5)	89.2% (74.6–97.0)	93.2% (89.3–96.1)	0.83 (0.76–0.89)
S2	Seton OR fistula procedure OR fistula/abscess/lesion	75.5% (61.1–86.7)	98.7% (96.2–99.7)	92.5% (79.6–98.4)	94.9% (91.2–97.3)	0.87 (0.81–0.93)
S5	(Seton OR fistula procedure OR fistula or abscess) OR (lesion excision OR hemorrhoid procedure AND perianal lesion)	75.8% (57.7–88.9)	98.5% (94.7–99.8)	92.6% (75.7–99.1)	94.2% (89.0–97.5)	0.87 (0.80–0.95)
S6	(Seton OR fistula procedure OR fistula/abscess/lesion) AND anti-TNFα	65.3% (50.4–78.3)	98.7% (96.2–99.7)	91.4% (76.9–98.2)	92.9% (88.9–95.8)	0.82 (0.75–0.89)

Cat, Category; PPV, positive predictive value; NPV, negative predictive value; ROC, receiver operating characteristic; TNFα, tumor necrosis factor alpha

The overall top performing case definition (J3) identified 42 patients (15%), of whom 4 were determined to be false positives through medical record review. This definition had a sensitivity of 77.6%, specificity 98.2%, PPV 90.5%, NPV 95.3%, and area under the ROC curve of 0.88.

## Discussion

We developed and evaluated novel alternative administrative claims-based methods to identify perianal fistula cases among pediatric patients with Crohn’s disease. We conducted a medical record review to evaluate the accuracy of an administrative claims-based case definition that has been used in prior studies.[6, 15, 16] In addition, we assessed a broad array of candidate case definitions that reflect the diversity of evaluation and treatment strategies commonly employed with Crohn’s disease. To our knowledge, this is the first claims-based method for identifying perianal fistulas among children with Crohn’s disease that has been validated with medical record review.

We found that several candidate definitions outperformed our reference case definition, which reflects a claims-based case definition used in prior studies. The most accurate case definition (J3) required codes indicating the presence of any perianal lesion (fistula, abscess, skin tag or hemorrhoid) or genital lesion (fistula or abscess) or any relevant surgical procedure (seton placement, fistulectomy, fistulotomy, hemorrhoidectomy, or removal of perianal lesion). This combination produced the most accurate result and identified an additional 88% of cases above that of the reference case definition in our study population. While several of our case definitions performed well, no case definition accurately identified all cases. Even our highest performing measures had some false negatives (affecting sensitivity) as well as false positives (affecting specificity). However, the highest-performing candidate case definitions identified fewer false negatives and false positives then the reference case definition, realizing an overall improvement in accuracy compared to prior claims-based methods.

Although no previous study has assessed the accuracy of claims definitions, administrative claims-based studies of perianal fistula have been performed among patients with Crohn’s disease. Prior claims-based studies have identified the prevalence of perianal fistulas ranging from 4–6%.[6, 15, 16] The few studies in which perianal fistulas were prospectively classified by physicians identified the rate of perianal fistulas which ranged from 15–27% among patients with Crohn’s disease diagnosis.[1, 12, 43–45] Our highest-performing case definitions indicated a prevalence of 15%, compared with a true prevalence of 18% among our patient population.

Our findings demonstrate that accurate claims-based methods for identifying perianal fistulizing disease among Crohn’s disease patients are feasible. We found several candidate measures that performed well, based on diagnosis, procedure, and medication codes readily available in administrative claims data. These claims-based case definitions can be broadly applied to administrative data widely available to commercial and Medicaid health plans. In contrast, prospective observational studies of perianal fistulas are difficult and time-consuming to conduct due to the requirement of performing perianal examination on patients who may not have active perianal complaints.[9, 43, 46] While there have been a limited number of prospective fistula treatment studies, they are based on small numbers of patients and cannot address epidemiology or healthcare utilization from a population perspective.[17, 22, 23, 25] Importantly, no study to date has addressed prevention of perianal fistulas.[47] We believe that our claims-based approach establishes a validated basis for identifying fistulizing Crohn’s cases, serving as the basis for future quality of care and outcomes studies.

While our claims-based alternative case definitions outperformed the reference case definition, there are still important limitations to acknowledge. First, the case definition of requiring a minimum of 3 Crohn’s disease claims used for identifying patients with Crohn’s disease is imperfect, and was likely to have identified patients without Crohn’s disease.[14, 32] However, we used medical record review to ensure that cases included in this study were confirmed to have Crohn’s disease. In addition, this definition has a high negative predictive value, giving a high degree of confidence that few cases of Crohn’s disease would be missed. Second, the medical record validation was performed at a single institution; it is possible that treatment and evaluation practices differ significantly at other institutions, which may affect the accuracy of the alternative case definitions. Practice differences may be substantially different at private practices or in underserved areas, or in different geographic locations. However, lacking any prior case definition validation findings (either the original ICD9 565.1 definition, or alternates), a single-institution patient-level study was needed to define the methods and establish the case definitions presented in this study.[48, 49] Additionally, this study was performed with patients enrolled in Medicaid; it is possible that patients with other forms of insurance or without insurance may have different evaluation or treatment patterns, which could affect the diagnostic accuracy of the case definitions in those settings. However, we designed our case definitions based on existing literature and expert opinion, representing current standards of care. While variation in treatment patterns certainly exist, the highest performing candidate case definitions included a broad range of treatment options encompassing the majority of documented practice patterns across populations including public and private insurance.[17, 26, 47, 50] One other limitation to acknowledge is that we excluded all events that occurred when patients were not enrolled in Medicaid. Consequently, it is likely that we missed some fistulas diagnosed outside the Medicaid enrollment period, which would bias our results to lower sensitivity. It is also important to note that our sample of patients with fistulas is relatively small; only 49 patients were identified to have perianal fistulas. Despite this, ours is the only study to date that evaluates the validity of claims-based definitions of perianal disease. Validation in other settings will strengthen our understanding of the reliability of these case definitions when they are applied to administrative claims for other populations.

Our claims-based method for identifying perianal disease among children with Crohn’s disease provides a validated method to identify patients for studies of prevention and treatment of perianal fistulas among children with Crohn’s disease. The high specificity observed in our best performing measures provides a high degree of confidence that cases identified using these methods are actually true cases of perianal fistulizing disease. Given the detrimental impact on quality of life and high cost-burden, interventions aimed at improving the quality care of patients with perianal fistula should now be feasible with these new methods.[2–6, 15] Consequently, we believe that these case definitions offer a mechanism to accurately identify pediatric patients with perianal Crohn’s disease using administrative claims data, enabling quality of care or comparative effectiveness studies. We believe that these validated methods provide the basis needed to evaluate care management approaches, which may ultimately lead to insights into effective mechanisms to lower rates of perianal fistulizing complications.

## Conclusions

We demonstrated that it is feasible to use administrative claims data to accurately identify pediatric patients with perianal fistula complications. Claims-based case definitions were found to be highly accurate through medical record review, providing a high degree of confidence for future studies where chart review is not feasible. These claims-based methods can be applied to claims data in other settings for the evaluation of health services utilization as well as to assess the comparative effectiveness of prevention and treatment strategies.

## Supporting information

S1 Table Claims classifications included in case definitions. (DOCX)

S2 Table Description of case definitions developed for identification of children with Crohn’s disease and perianal fistula from claims. (DOCX)

S3 Table Frequencies of perianal fistula by case definition applied to Crohn’s disease patients. (DOCX)

S4 Table Measures of performance for case definitions applied to Crohn’s disease patients. (DOCX)

S5 Table Minimal data set containing de-identified patient-level data. (XLS)

S1 Checklist The REporting of studies conducted using observational routinely-collected health Data (RECORD) checklist. (DOCX)
