# Supplementary material for: Identifying perianal fistula complications in pediatric patients with Crohn’s disease using administrative claims
Source: PLoS One. 2019 Aug 14;14(8):e0219893. doi: 10.1371/journal.pone.0219893 (PMC6693740; doi:10.1371/journal.pone.0219893)
Supplement: S1 Table — (DOCX) [file pone.0219893.s001.docx]

**S1 Table. Claims Classifications Included in Case Definitions**

| Category | Definition* |
| --- | --- |
| Perianal fistula | 565.1 |
| Genital fistula | 619.1, 619.2, 619.8, 619.9, 607.89, 599.1 |
| Perirectal abscess | 566.x, 682.5 |
| Genital abscess | 616.4, 601.2, 593.89, 597.0, 595.89, 608.4 |
| Perianal lesions | 455.9, 569.49, 455.x |
| Genital lesions | 607.89, 614.4, 707.9, 616.10, 616.9, 624.8 |
| Anti-TNFα medication | Infliximab, adalimumab, certolizumab |
| Immunomodulator | Azathioprine, 6-mercaptopurine, methotrexate |
| Antibiotic | Metronidazole, ciprofloxacin, levofloxacin |
| Seton | CPT: 46020-51 |
| Fistulotomy | ICD-9: 49.11  CPT: 46270, 46275, 46280, 46285 |
| Fistulectomy | ICD-9: 49.12  CPT: 46270, 46275, 46280, 46285, 46262 |
| I&D perianal abscess | ICD-9: 49.1, 49.01  CPT: 46040, 46045-51, 46060, 46000, 46005, 45020 |
| Removal perianal lesion | ICD-9: 49.02, 49.03, 49.04, 49.39  CPT: 46220, 46230, 46900, 46910, 46916, 46917, 46922, 46924 |
| Hemorrhoid procedure | ICD-9: 49.45, 49.46  CPT: 46221, 46230, 46250, 46255, 46257, 46258, 46260, 46261, 46262, 46320, 46934, 46935, 46936, 46500 |
| Perianal fistula closure | ICD-9: 49.73  CPT: 46270, 46275, 46280, 46285, 46288, 46706, 46707, 46280 |

*Numbers represent International Classification of Diseases version 9 (ICD-9) unless otherwise specified. I&D, incision and drainage; CPT, Current Procedural Terminology; TNFα, tumor necrosis factor alpha
