# Supplementary material for: Identifying perianal fistula complications in pediatric patients with Crohn’s disease using administrative claims
Source: PLoS One. 2019 Aug 14;14(8):e0219893. doi: 10.1371/journal.pone.0219893 (PMC6693740; doi:10.1371/journal.pone.0219893)
Supplement: S2 Table — (DOCX) [file pone.0219893.s002.docx]

**S2 Table. Description of Case Definitions Developed for Identification of Children with Crohn’s disease and Perianal Fistula from Claims**

| **Case Definition** | **Criteria** |
| --- | --- |
| **Reference case definition** |  |
| A1 | Perianal fistula 565.1 |
| **Perianal fistula** (composite) |  |
| B1 | Perianal fistula OR perirectal abscess |
| B2 | Perianal fistula OR perirectal abscess OR genital fistula OR genital abscess |
| B3 | Perianal fistula OR perirectal abscess OR genital fistula OR genital abscess OR perianal lesion |
| **Medication only**  (no perianal lesion) |  |
| C1 | Anti-TNFα |
| C2 | Anti-TNFα AND antibiotic |
| C3 | Anti-TNFα OR antibiotic |
| C4 | Immunomodulator AND anti-TNFα |
| C5 | Immunomodulator AND anti-TNFα AND antibiotic |
| C6 | Immunomodulator OR anti-TNFα |
| C7 | (Immunomodulator OR anti-TNFα) AND antibiotic |
| **Perianal fistula AND medication** |  |
| D1 | Perianal fistula AND anti-TNFα |
| D2 | Perianal fistula AND anti-TNFα AND antibiotic |
| D3 | Perianal fistula AND (anti-TNFα OR antibiotic) |
| D4 | Perianal fistula AND Immunomodulator |
| D5 | Perianal fistula AND Immunomodulator AND antibiotic |
| **Perianal fistula/lesion AND medication** |  |
| E1 | Perianal fistula/lesion AND anti-TNFα |
| E2 | Perianal fistula/lesion AND anti-TNFα AND antibiotic |
| E3 | Perianal fistula/lesion AND (anti-TNFα OR antibiotic) |
| E4 | Perianal fistula/lesion AND immunomodulator |
| E5 | Perianal fistula/lesion AND immunomodulator AND antibiotic |
| **Procedure** |  |
| F1 | Seton |
| F2 | Excision of lesion |
| F3 | Fistula closure |
| F4 | Hemorrhoid procedure |
| F5 | Excision OR hemorrhoid procedure |
| F6 | Fistula, excision OR hemorrhoid procedure |
| F7 | Seton OR fistula procedure |
| F8 | Seton, fistula, excision OR hemorrhoid procedure |
| **Procedure AND perianal lesion** |  |
| G1 | Seton AND perianal lesion |
| G2 | Excision of lesion AND perianal lesion |
| G3 | Fistula closure AND perianal lesion |
| G4 | Hemorrhoid procedure AND perianal lesion |
| G5 | Excision OR hemorrhoid procedure AND perianal lesion |
| G6 | Fistula, excision OR hemorrhoid procedure AND perianal lesion |
| G7 | Seton, fistula, excision OR hemorrhoid procedure AND perianal lesion |
| **Procedure AND perianal fistula** |  |
| H1 | Seton AND perianal fistula |
| H2 | Excision of lesion AND perianal fistula |
| H3 | Fistula closure AND perianal fistula |
| H4 | Hemorrhoid procedure AND perianal fistula |
| H5 | Excision OR hemorrhoid procedure AND perianal fistula |
| H6 | Fistula, excision OR hemorrhoid procedure AND perianal fistula |
| H7 | Seton, fistula, excision OR hemorrhoid procedure AND perianal fistula |
| **Procedure AND perianal fistula/lesion** |  |
| I1 | Seton AND perianal fistula/lesion |
| I2 | Excision of lesion AND perianal fistula/lesion |
| I3 | Fistula closure AND perianal fistula/lesion |
| I4 | Hemorrhoid procedure AND perianal fistula/lesion |
| I5 | (Excision OR hemorrhoid procedure) AND perianal fistula/lesion |
| I6 | (Fistula, excision OR hemorrhoid procedure) AND perianal fistula/lesion |
| I7 | (Seton, fistula, excision OR hemorrhoid procedure) AND perianal fistula/lesion |
| **Procedure OR perianal fistula/lesion** |  |
| J1 | (Seton, fistula closure/excision, hemorrhoid procedure) OR perianal fistula/lesion |
| J2 | (Seton, fistula closure/excision, hemorrhoid procedure) OR perianal/genital fistula/abscess |
| J3 | (Seton, fistula closure/excision, hemorrhoid procedure) OR perianal/genital fistula/abscess/lesion |
| **Imaging** |  |
| K1 | CT |
| K2 | MRI |
| K3 | Fluoroscopy |
| K4 | EUS |
| K5 | CT OR MRI |
| **Imaging AND perianal lesion** |  |
| L1 | CT AND perianal lesion |
| L2 | MRI AND perianal lesion |
| L3 | Fluoroscopy AND perianal lesion |
| L4 | EUS AND perianal lesion |
| L5 | (CT OR MRI) AND perianal lesion |
| **Imaging AND perianal fistula** |  |
| M1 | CT AND perianal fistula |
| M2 | MRI AND perianal fistula |
| M3 | Fluoroscopy AND perianal fistula |
| M4 | EUS AND perianal fistula |
| M5 | (CT OR MRI) AND perianal fistula |
| **Imaging AND perianal fistula/lesion** |  |
| N1 | CT AND perianal fistula/lesion |
| N2 | MRI AND perianal fistula/lesion |
| N3 | Fluoroscopy AND perianal fistula/lesion |
| N4 | EUS AND perianal fistula/lesion |
| N5 | (CT OR MRI) AND perianal fistula/lesion |
| **Imaging AND medication** |  |
| O1 | (CT OR MRI) AND anti-TNFα |
| O2 | (CT OR MRI) AND anti-TNFα AND antibiotic |
| O3 | (CT OR MRI) AND (anti-TNFα OR antibiotic) |
| O4 | (CT OR MRI) AND immunomodulator |
| O5 | (CT OR MRI) AND immunomodulator AND antibiotic |
| **Imaging AND perianal lesion AND medication** |  |
| P1 | (CT OR MRI) AND perianal lesion AND anti-TNFα |
| P2 | (CT OR MRI) AND perianal lesion AND anti-TNFα AND antibiotic |
| P3 | (CT OR MRI) AND perianal lesion AND (anti-TNFα OR antibiotic) |
| P4 | (CT OR MRI) AND perianal lesion AND immunomodulator |
| P5 | (CT OR MRI) AND perianal lesion AND immunomodulator AND antibiotic |
| **Imaging AND perianal fistula AND medication** |  |
| Q1 | (CT OR MRI) AND perianal fistula AND anti-TNFα |
| Q2 | (CT OR MRI) AND perianal fistula AND anti-TNFα AND antibiotic |
| Q3 | (CT OR MRI) AND perianal fistula AND (anti-TNFα OR antibiotic) |
| Q4 | (CT OR MRI) AND perianal fistula AND immunomodulator |
| Q5 | (CT OR MRI) AND perianal fistula AND immunomodulator AND antibiotic |
| **Imaging AND perianal fistula/lesion AND medication** |  |
| R1 | (CT OR MRI) AND perianal fistula/lesion AND Anti-TNFα |
| R2 | (CT OR MRI) AND perianal fistula/lesion AND Anti-TNFα AND antibiotic |
| R3 | (CT OR MRI) AND perianal fistula/lesion AND (anti-TNFα OR antibiotic) |
| R4 | (CT OR MRI) AND perianal fistula/lesion AND immunomodulator |
| R5 | (CT OR MRI) AND perianal fistula/lesion AND immunomodulator AND antibiotic |
| **Other combinations** |  |
| S1 | Seton OR fistula procedure OR fistula or abscess |
| S2 | Seton OR fistula procedure OR fistula/abscess/lesion |
| S3 | (Seton OR fistula procedure OR EUS) and (fistula/abscess/lesion) |
| S4 | (Seton OR fistula procedure OR EUS or MRI or CT) and (fistula/abscess/lesion) and antibiotic |
| S5 | (Seton OR fistula procedure OR fistula or abscess) OR G4 OR G5 |
| S6 | (Seton OR fistula procedure OR fistula/abscess/lesion) AND anti-TNFα |

Anti-TNFα, anti-tumor necrosis factor alpha medication; CT, computed tomography imaging; MRI, magnetic resonance imaging; EUS, endoscopic ultrasound
