# Supplementary material for: Identifying perianal fistula complications in pediatric patients with Crohn’s disease using administrative claims
Source: PLoS One. 2019 Aug 14;14(8):e0219893. doi: 10.1371/journal.pone.0219893 (PMC6693740; doi:10.1371/journal.pone.0219893)
Supplement: S3 Table — (DOCX) [file pone.0219893.s003.docx]

**S3 Table. Frequencies of Perianal Fistula by Case Definition Applied to Crohn’s Disease Patients**

| **Definition Category** | **Category** | **Number** | **True**  **Positive** | **False**  **Negative** | **False**  **Positive** | **True**  **Negative** |
| --- | --- | --- | --- | --- | --- | --- |
| A1 | **Reference case definition** | 274 | 25 | 24 | 1 | 224 |
| B1 | **Perianal fistula** | 274 | 33 | 16 | 1 | 224 |
| B2 | (composite) | 274 | 31 | 18 | 1 | 224 |
| **B3** |  | **274** | **35** | **14** | **1** | **224** |
| C1 | **Medication only** | 274 | 43 | 6 | 113 | 112 |
| **C2** | (no perianal lesion) | **166** | **26** | **7** | **38** | **95** |
| C3 |  | 166 | 32 | 1 | 104 | 29 |
| C4 |  | 166 | 19 | 14 | 50 | 83 |
| C5 |  | 166 | 19 | 14 | 36 | 97 |
| C6 |  | 166 | 31 | 2 | 105 | 28 |
| C7 |  | 166 | 26 | 7 | 58 | 75 |
| D1 | **Perianal fistula AND** | 274 | 23 | 26 | 1 | 224 |
| D2 | **medication** | 166 | 15 | 18 | 0 | 133 |
| **D3** |  | **166** | **19** | **14** | **0** | **133** |
| D4 |  | 166 | 13 | 20 | 0 | 133 |
| D5 |  | 166 | 9 | 24 | 0 | 133 |
| E1 | **Perianal fistula/lesion AND** | 274 | 27 | 22 | 1 | 224 |
| E2 | **medication** | 166 | 20 | 13 | 1 | 132 |
| **E3** |  | **166** | **24** | **9** | **1** | **132** |
| E4 |  | 166 | 14 | 19 | 1 | 132 |
| E5 |  | 166 | 13 | 20 | 1 | 132 |
| F1 | **Procedure** | 274 | 7 | 42 | 3 | 222 |
| F2 |  | 274 | 3 | 46 | 0 | 225 |
| F3 |  | 274 | 6 | 43 | 1 | 224 |
| F4 |  | 274 | 1 | 48 | 1 | 224 |
| F5 |  | 274 | 3 | 46 | 1 | 224 |
| F6 |  | 274 | 9 | 40 | 2 | 223 |
| F7 |  | 274 | 8 | 41 | 3 | 222 |
| **F8** |  | **274** | **11** | **38** | **4** | **221** |
| G1 | **Procedure AND perianal** | 274 | 3 | 46 | 1 | 224 |
| G2 | **lesion** | 274 | 3 | 46 | 0 | 225 |
| G3 |  | 274 | 2 | 47 | 0 | 225 |
| G4 |  | 274 | 1 | 48 | 0 | 225 |
| G5 |  | 274 | 3 | 46 | 0 | 225 |
| G6 |  | 274 | 5 | 44 | 0 | 225 |
| **G7** |  | **274** | **6** | **43** | **1** | **224** |
| H1 | **Procedure AND perianal** | 274 | 5 | 44 | 0 | 225 |
| H2 | **fistula** | 274 | 1 | 48 | 0 | 225 |
| H3 |  | 274 | 4 | 45 | 0 | 225 |
| H4 |  | 274 | 1 | 48 | 0 | 225 |
| H5 |  | 274 | 1 | 48 | 0 | 225 |
| H6 |  | 274 | 5 | 44 | 0 | 225 |
| **H7** |  | **274** | **7** | **42** | **0** | **225** |
| I1 | **Procedure AND perianal** | 274 | 5 | 44 | 1 | 224 |
| I2 | **fistula/lesion** | 274 | 2 | 47 | 0 | 225 |
| I3 |  | 274 | 4 | 45 | 0 | 225 |
| I4 |  | 274 | 1 | 48 | 0 | 225 |
| I5 |  | 274 | 2 | 47 | 0 | 225 |
| I6 |  | 274 | 6 | 43 | 0 | 225 |
| **I7** |  | **274** | **8** | **41** | **1** | **224** |
| J1 | **Procedure OR perianal** | 274 | 33 | 16 | 4 | 221 |
| J2 | **fistula/lesion** | 274 | 36 | 13 | 5 | 220 |
| **J3** |  | **274** | **38** | **11** | **4** | **221** |
| K1 | **Imaging** | 274 | 20 | 29 | 100 | 125 |
| **K2** |  | **274** | **37** | **12** | **69** | **156** |
| K3 |  | 274 | 3 | 46 | 19 | 206 |
| K4 |  | 274 | 0 | 49 | 0 | 225 |
| K5 |  | 274 | 41 | 8 | 136 | 89 |
| L1 | **Imaging AND perianal** | 274 | 7 | 42 | 1 | 224 |
| L2 | **lesion** | 274 | 13 | 36 | 1 | 224 |
| L3 |  | 274 | 1 | 48 | 0 | 225 |
| L4 |  | 274 | 0 | 49 | 0 | 225 |
| **L5** |  | **274** | **14** | **35** | **1** | **224** |
| M1 | **Imaging AND perianal** | 274 | 18 | 31 | 1 | 224 |
| M2 | **fistula** | 274 | 26 | 23 | 1 | 224 |
| M3 |  | 274 | 3 | 46 | 1 | 224 |
| M4 |  | 274 | 0 | 49 | 0 | 225 |
| **M5** |  | **274** | **30** | **19** | **1** | **224** |
| N1 | **Imaging AND perianal** | 274 | 18 | 31 | 1 | 224 |
| N2 | **fistula/lesion** | 274 | 27 | 22 | 1 | 224 |
| N3 |  | 274 | 3 | 46 | 0 | 225 |
| N4 |  | 274 | 0 | 49 | 0 | 225 |
| **N5** |  | **274** | **31** | **18** | **1** | **224** |
| **O1** | **Imaging AND medication** | **274** | **35** | **14** | **80** | **145** |
| O2 |  | 166 | 22 | 11 | 33 | 100 |
| O3 |  | 166 | 28 | 5 | 69 | 64 |
| O4 |  | 166 | 16 | 17 | 54 | 79 |
| O5 |  | 166 | 15 | 18 | 41 | 92 |
| **P1** | **Imaging AND perianal** | **274** | **14** | **35** | **1** | **224** |
| P2 | **lesion AND medication** | 166 | 11 | 22 | 1 | 132 |
| P3 |  | 166 | 12 | 21 | 1 | 132 |
| P4 |  | 166 | 8 | 25 | 1 | 132 |
| P5 |  | 166 | 8 | 25 | 1 | 132 |
| Q1 | **Imaging AND perianal** | 274 | 26 | 23 | 1 | 224 |
| Q2 | **fistula AND medication** | 166 | 17 | 16 | 0 | 133 |
| **Q3** |  | **166** | **22** | **11** | **0** | **133** |
| Q4 |  | 166 | 10 | 23 | 0 | 133 |
| Q5 |  | 166 | 10 | 23 | 0 | 133 |
| R1 | **Imaging AND perianal** | 274 | 27 | 22 | 1 | 224 |
| R2 | **fistula/lesion AND** | 274 | 18 | 31 | 1 | 224 |
| **R3** | **medication** | **274** | **30** | **19** | **1** | **224** |
| R4 |  | 166 | 12 | 21 | 1 | 132 |
| R5 |  | 166 | 11 | 22 | 1 | 132 |
| S1 | **Other combinations** | 274 | 33 | 16 | 4 | 221 |
| **S2** |  | **274** | **37** | **12** | **3** | **222** |
| S3 |  | 274 | 5 | 44 | 1 | 224 |
| S4 |  | 166 | 20 | 13 | 1 | 132 |
| S5 |  | 166 | 25 | 8 | 2 | 131 |
| S6 |  | 274 | 32 | 17 | 3 | 222 |

Shading represents definition categories. **Bold** text represents the best performing case definition per category.
