# Supplementary material for: Identifying perianal fistula complications in pediatric patients with Crohn’s disease using administrative claims
Source: PLoS One. 2019 Aug 14;14(8):e0219893. doi: 10.1371/journal.pone.0219893 (PMC6693740; doi:10.1371/journal.pone.0219893)
Supplement: S4 Table — (DOCX) [file pone.0219893.s004.docx]

**S4 Table. Measures of Performance for Case Definitions**

| **Definition Category** | **Category** | **Sensitivity**  **(95% CI)** | **Specificity**  **(95% CI)** | **PPV**  **(95% CI)** | **NPV**  **(95% CI)** | **Area Under ROC Curve (95% CI)** |
| --- | --- | --- | --- | --- | --- | --- |
| A1 | **Reference case definition** | 51.0% (36.3-65.6) | 99.6% (97.5-100.0) | 96.2% (80.4-99.9) | 90.3% (85.9-93.7) | 0.75 (0.68-0.82) |
| B1 | **Perianal fistula** | 67.3% (52.5-80.1) | 99.6% (97.5-100.0) | 97.1% (84.7-99.9) | 93.3% (89.4-96.1) | 0.83 (0.77-0.90) |
| B2 | (composite) | 63.3% (48.3-76.6) | 99.6% (97.5-100.0) | 96.9% (83.8-99.9) | 92.6% (88.5-95.5) | 0.81 (0.75-0.88) |
| **B3** |  | **71.4% (56.7-83.4)** | **99.6% (97.5-100.0)** | **97.2% (85.5-99.9)** | **94.1% (90.3-96.7)** | **0.85 (0.79-0.92)** |
| C1 | **Medication only** | 87.8% (75.2-95.4) | 49.8% (43.1-56.5) | 27.6% (20.7-35.3) | 94.9% (89.3-98.1) | 0.69 (0.63-0.74) |
| **C2** | (no perianal lesion) | **79.6% (65.7-89.8)** | **63.6% (56.9-69.8)** | **32.2% (24.0-41.3)** | **93.5% (88.3-96.8)** | **0.72 (0.65-0.78)** |
| C3 |  | 98.0% (89.1-99.9) | 12.9% (8.8-18.0) | 19.7% (14.9-25.2) | 96.7% (82.8-99.9) | 0.55 (0.52-0.58) |
| C4 |  | 98.0% (89.1-99.9) | 12.9% (8.8-18.0) | 19.7% (14.9-25.2) | 96.7% (82.8-99.9) | 0.55 (0.52-0.58) |
| C5 |  | 98.0% (89.1-99.9) | 12.9% (8.8-18.0) | 19.7% (14.9-25.2) | 96.7% (82.8-99.9) | 0.55 (0.52-0.58) |
| C6 |  | 95.9% (86.0-99.5) | 12.4% (8.4-17.5) | 19.3% (14.5-24.8) | 93.3% (77.9-99.2) | 0.54 (0.51-0.58) |
| C7 |  | 85.7% (72.8-94.1) | 33.3% (27.2-39.9) | 21.9% (16.2-28.4) | 91.5% (83.2-96.5) | 0.60 (0.54-0.65) |
| D1 | **Perianal fistula AND** | 46.9% (32.5-61.7) | 99.6% (97.5-100.0) | 95.8% (78.9-99.9) | 89.6% (85.1-93.1) | 0.73 (0.66-0.80) |
| D2 | **medications** | 40.8% (27.0-55.8) | 99.6% (97.5-100.0) | 95.2% (76.2-99.9) | 88.5% (84.0-92.2) | 0.70 (0.63-0.77) |
| **D3** |  | **49.0% (34.4-63.7)** | **99.6% (97.5-100.0)** | **96.0% (79.6-99.9)** | **90.0% (85.5-93.4)** | **0.74 (0.67-0.81)** |
| D4 |  | 26.5% (14.9-41.1) | 100% (98.4-100.0) | 100% (75.3-100.0) | 86.2% (81.4-90.1) | 0.63 (0.57-0.70) |
| D5 |  | 30.6% (18.3-45.4) | 99.6% (97.5-100.0) | 93.8% (69.8-99.8) | 86.8% (82.1-90.7) | 0.65 (0.59-0.72) |
| E1 | **Perianal fistula/lesion** | 55.1% (40.2-69.3) | 99.6% (97.5-100.0) | 96.4% (81.7-99.9) | 91.1% (86.8-94.3) | 0.77 (0.70-0.84) |
| E2 | **AND medications** | 49.0% (34.4-63.7) | 99.6% (97.5-100.0) | 96.0% (79.6-99.9) | 90.0% (85.5-93.4) | 0.74 (0.67-0.81) |
| **E3** |  | **59.2% (44.2-73.0)** | **99.6% (97.5-100.0)** | **96.7% (82.8-99.9)** | **91.8% (87.6-94.9)** | **0.79 (0.72-0.86)** |
| E4 |  | 38.8% (25.2-53.8) | 99.6% (97.5-100.0) | 95.0% (75.1-99.9) | 88.2% (83.6-91.9) | 0.69 (0.62-0.76) |
| E5 |  | 36.7% (23.4-51.7) | 99.6% (97.5-100.0) | 94.7% (74.0-99.9) | 87.8% (83.2-91.6) | 0.68 (0.61-0.75) |
| F1 | **Procedure** | 14.3% (5.9-27.2) | 98.7% (96.2-99.7) | 70.0% (34.8-93.3) | 84.1% (79.1-88.3) | 0.56 (0.51-0.61) |
| F2 |  | 6.1% (1.3-16.9) | 100% (98.4-100.0) | 100% (29.2-100.0) | 83.0% (78.0-87.3) | 0.53 (0.50-0.56) |
| F3 |  | 12.2% (4.6-24.8) | 99.6% (97.5-100.0) | 85.7% (42.1-99.6) | 83.9% (78.9-88.1) | 0.56 (0.51-0.61) |
| F4 |  | 2.0% (0.1-10.9) | 99.6% (97.5-100.0) | 50.0% (1.3-98.7) | 82.4% (77.3-86.7) | 0.51 (0.49-0.53) |
| F5 |  | 6.1% (1.3-16.9) | 99.6% (97.5-100.0) | 75.0% (19.4-99.4) | 83.0% (77.9-87.2) | 0.53 (0.49-0.56) |
| F6 |  | 18.4% (8.8-32.0) | 99.1% (96.8-99.9) | 81.8% (48.2-97.7) | 84.8% (79.9-88.9) | 0.59 (0.53-0.64) |
| F7 |  | 16.3% (7.3-29.7) | 98.7% (96.2-99.7) | 72.7% (39.0-94.0) | 84.4% (79.5-88.6) | 0.57 (0.52-0.63) |
| **F8** |  | **22.4% (11.8-36.6)** | **98.2% (95.5-99.5)** | **73.3% (44.9-92.2)** | **85.3% (80.4-89.4)** | **0.60 (0.54-0.66)** |
| G1 | **Procedure AND** | 6.1% (1.3-16.9) | 99.6% (97.5-100.0) | 75.0% (19.4-99.4) | 83.0% (77.9-87.2) | 0.53 (0.49-0.56) |
| G2 |  | 6.1% (1.3-16.9) | 100% (98.4-100.0) | 100% (29.2-100.0) | 83.0% (78.0-87.3) | 0.53 (0.50-0.56) |
| G3 |  | 4.1% (0.5-14.0) | 100% (98.4-100.0) | 100% (15.8-100.0) | 82.7% (77.7-87.0) | 0.52 (0.49-0.55) |
| G4 |  | 2.0% (0.1-10.9) | 100% (98.4-100.0) | 100% (2.5-100.0) | 82.4% (77.4-86.7) | 0.51 (0.49-0.53) |
| G5 |  | 6.1% (1.3-16.9) | 100% (98.4-100.0) | 100% (29.2-100.0) | 83.0% (78.0-87.3) | 0.53 (0.50-0.56) |
| G6 |  | 10.2% (3.4-22.2) | 100% (98.4-100.0) | 100% (47.8-100.0) | 83.6% (78.7-87.9) | 0.55 (0.51-0.59) |
| **G7** |  | **12.2% (4.6-24.8)** | **99.6% (97.5-100.0)** | **85.7% (42.1-99.6)** | **83.9% (78.9-88.1)** | **0.56 (0.51-0.61)** |
| H1 | **Procedure AND** | 10.2% (3.4-22.2) | 100% (98.4-100.0) | 100% (47.8-100.0) | 83.6% (78.7-87.9) | 0.55 (0.51-0.59) |
| H2 | **perianal fistula** | 2.0% (0.1-10.9) | 100% (98.4-100.0) | 100% (2.5-100.0) | 82.4% (77.4-86.7) | 0.51 (0.49-0.53) |
| H3 |  | 8.2% (2.3-19.6) | 100% (98.4-100.0) | 100% (39.8-100.0) | 83.3% (78.3-87.6) | 0.54 (0.50-0.58) |
| H4 |  | 2.0% (0.1-10.9) | 100% (98.4-100.0) | 100% (2.5-100.0) | 82.4% (77.4-86.7) | 0.51 (0.49-0.53) |
| H5 |  | 2.0% (0.1-10.9) | 100% (98.4-100.0) | 100% (2.5-100.0) | 82.4% (77.4-86.7) | 0.51 (0.49-0.53) |
| H6 |  | 10.2% (3.4-22.2) | 100% (98.4-100.0) | 100% (47.8-100.0) | 83.6% (78.7-87.9) | 0.55 (0.51-0.59) |
| **H7** |  | **14.3% (5.9-27.2)** | **100% (98.4-100.0)** | **100% (59.0-100.0)** | **84.3% (79.3-88.4)** | **0.57 (0.52-0.62)** |
| I1 | **Procedure AND** | 10.2% (3.4-22.2) | 99.6% (97.5-100.0) | 83.3% (35.9-99.6) | 83.6% (78.6-87.8) | 0.55 (0.51-0.59) |
| I2 | **perianal fistula/** | 4.1% (0.5-14.0) | 100% (98.4-100.0) | 100% (15.8-100.0) | 82.7% (77.7-87.0) | 0.52 (0.49-0.55) |
| I3 | **lesion** | 8.2% (2.3-19.6) | 100% (98.4-100.0) | 100% (39.8-100.0) | 83.3% (78.3-87.6) | 0.54 (0.50-0.58) |
| I4 |  | 2.0% (0.1-10.9) | 100% (98.4-100.0) | 100% (2.5-100.0) | 82.4% (77.4-86.7) | 0.51 (0.49-0.53) |
| I5 |  | 4.1% (0.5-14.0) | 100% (98.4-100.0) | 100% (15.8-100.0) | 82.7% (77.7-87.0) | 0.52 (0.49-0.55) |
| I6 |  | 12.2% (4.6-24.8) | 100% (98.4-100.0) | 100% (54.1-100.0) | 84.0% (79.0-88.1) | 0.56 (0.51-0.61) |
| **I7** |  | **16.3% (7.3-29.7)** | **99.6% (97.5-100.0)** | **88.9% (51.8-99.7)** | **84.5% (79.6-88.7)** | **0.58 (0.53-0.63)** |
| J1 | **Procedure OR** | 67.3% (52.5-80.1) | 98.2% (95.5-99.5) | 89.2% (74.6-97.0) | 93.2% (89.3-96.1) | 0.83 (0.76-0.89) |
| J2 | **perianal fistula/** | 73.5% (58.9-85.1) | 97.8% (94.9-99.3) | 87.8% (73.8-95.9) | 94.4% (90.6-97.0) | 0.86 (0.79-0.92) |
| **J3** | **lesion** | **77.6% (63.4-88.2)** | **98.2% (95.5-99.5)** | **90.5% (77.4-97.3)** | **95.3% (91.7-97.6)** | **0.88 (0.82-0.94)** |
| K1 | **Imaging** | 40.8% (27.0-55.8) | 55.6% (48.8-62.2) | 16.7% (10.5-24.6) | 81.2% (74.1-87.0) | 0.48 (0.41-0.56) |
| **K2** |  | **75.5% (61.1-86.7)** | **69.3% (62.9-75.3)** | **34.9% (25.9-44.8)** | **92.9% (87.9-96.3)** | **0.72 (0.66-0.79)** |
| K3 |  | 6.1% (1.3-16.9) | 91.6% (87.1-94.8) | 13.6% (2.9-34.9) | 81.7% (76.4-86.3) | 0.49 (0.45-0.53) |
| K4* |  | - | - | - | - | - |
| K5 |  | 83.7% (70.3-92.7) | 39.6% (33.1-46.3) | 23.2% (17.2-30.1) | 91.8% (84.4-96.4) | 0.62 (0.55-0.68) |
| L1 | **Imaging AND** | 14.3% (5.9-27.2) | 99.6% (97.5-100.0) | 87.5% (47.3-99.7) | 84.2% (79.3-88.4) | 0.57 (0.52-0.62) |
| L2 | **perianal lesion** | 26.5% (14.9-41.1) | 99.6% (97.5-100.0) | 92.9% (66.1-99.8) | 86.2% (81.3-90.1) | 0.63 (0.57-0.69) |
| L3 |  | 2.0% (0.1-10.9) | 100% (98.4-100.0) | 100% (2.5-100.0) | 82.4% (77.4-86.7) | 0.51 (0.49-0.53) |
| L4* |  | - | - | - | - | - |
| **L5** |  | **28.6% (16.6-43.3)** | **99.6% (97.5-100.0)** | **93.3% (68.1-99.8)** | **86.5% (81.7-90.4)** | **0.64 (0.58-0.70)** |
| M1 | **Imaging AND** | 36.7% (23.4-51.7) | 99.6% (97.5-100.0) | 94.7% (74.0-99.9) | 87.8% (83.2-91.6) | 0.68 (0.61-0.75) |
| M2 | **perianal fistula** | 53.1% (38.3-67.5) | 99.6% (97.5-100.0) | 96.3% (81.0-99.9) | 90.7% (86.4-94.0) | 0.76 (0.69-0.83) |
| M3 |  | 6.1% (1.3-16.9) | 99.6% (97.5-100.0) | 75.0% (19.4-99.4) | 83.0% (77.9-87.2) | 0.53 (0.49-0.56) |
| M4 |  | - | - | - | - | - |
| **M5** |  | **61.2% (46.2-74.8)** | **99.6% (97.5-100.0)** | **96.8% (83.3-99.9)** | **92.2% (88.1-95.2)** | **0.80 (0.73-0.87)** |
| N1 | **Imaging AND** | 36.7% (23.4-51.7) | 99.6% (97.5-100.0) | 94.7% (74.0-99.9) | 87.8% (83.2-91.6) | 0.68 (0.61-0.75) |
| N2 | **perianal fistula/** | 55.1% (40.2-69.3) | 99.6% (97.5-100.0) | 96.4% (81.7-99.9) | 91.1% (86.8-94.3) | 0.77 (0.70-0.84) |
| N3 | **lesion** | 6.1% (1.3-16.9) | 100% (98.4-100.0) | 100% (29.2-100.0) | 83.0% (78.0-87.3) | 0.53 (0.50-0.56) |
| N4* |  | - | - | - | - | - |
| **N5** |  | **63.3% (48.3-76.6)** | **99.6% (97.5-100.0)** | **96.9% (83.8-99.9)** | **92.6% (88.5-95.5)** | **0.81 (0.75-0.88)** |
| **O1** | **Imaging AND** | **71.4% (56.7-83.4)** | **64.4% (57.8-70.7)** | **30.4% (22.2-39.7)** | **91.2% (85.7-95.1)** | **0.68 (0.61-0.75)** |
| O2 | **medication** | 44.9% (30.7-59.8) | 84.9% (79.5-89.3) | 39.3% (26.5-53.2) | 87.6% (82.5-91.7) | 0.65 (0.57-0.72) |
| O3 |  | 81.6% (68.0-91.2) | 44.0% (37.4-50.8) | 24.1% (17.8-31.3) | 91.7% (84.8-96.1) | 0.63 (0.56-0.69) |
| O4 |  | 32.7% (19.9-47.5) | 75.6% (69.4-81.0) | 22.5% (13.5-34.0) | 83.7% (77.9-88.5) | 0.54 (0.47-0.61) |
| O5 |  | 30.6% (18.3-45.4) | 81.3% (75.6-86.2) | 26.3% (15.5-39.7) | 84.3% (78.8-88.9) | 0.56 (0.49-0.63) |
| **P1** | **Imaging AND** | **28.6% (16.6-43.3)** | **99.6% (97.5-100.0)** | **93.3% (68.1-99.8)** | **86.5% (81.7-90.4)** | **0.64 (0.58-0.70)** |
| P2 | **perianal lesion** | 22.4% (11.8-36.6) | 99.6% (97.5-100.0) | 91.7% (61.5-99.8) | 85.5% (80.6-89.5) | 0.61 (0.55-0.67) |
| P3 | **AND medication** | 28.6% (16.6-43.3) | 99.6% (97.5-100.0) | 93.3% (68.1-99.8) | 86.5% (81.7-90.4) | 0.64 (0.58-0.70) |
| P4 |  | 16.3% (7.3-29.7) | 99.6% (97.5-100.0) | 88.9% (51.8-99.7) | 84.5% (79.6-88.7) | 0.58 (0.53-0.63) |
| P5 |  | 16.3% (7.3-29.7) | 99.6% (97.5-100.0) | 88.9% (51.8-99.7) | 84.5% (79.6-88.7) | 0.58 (0.53-0.63) |
| Q1 | **Imaging AND perianal** | 53.1% (38.3-67.5) | 99.6% (97.5-100.0) | 96.3% (81.0-99.9) | 90.7% (86.4-94.0) | 0.76 (0.69-0.83) |
| Q2 | **fistula AND** | 34.7% (21.7-49.6) | 100% (98.4-100.0) | 100% (80.5-100.0) | 87.5% (82.9-91.3) | 0.67 (0.61-0.74) |
| **Q3** | **medication** | **59.2% (44.2-73.0)** | **99.6% (97.5-100.0)** | **96.7% (82.8-99.9)** | **91.8% (87.6-94.9)** | **0.79 (0.72-0.86)** |
| Q4 |  | 20.4% (10.2-34.3) | 100% (98.4-100.0) | 100% (69.2-100.0) | 85.2% (80.4-89.3) | 0.60 (0.55-0.66) |
| Q5 |  | 20.4% (10.2-34.3) | 100% (98.4-100.0) | 100% (69.2-100.0) | 85.2% (80.4-89.3) | 0.60 (0.55-0.66) |
| R1 | **Imaging AND perianal** | 55.1% (40.2-69.3) | 99.6% (97.5-100.0) | 96.4% (81.7-99.9) | 91.1% (86.8-94.3) | 0.77 (0.70-0.84) |
| R2 | **fistula/lesion AND** | 36.7% (23.4-51.7) | 99.6% (97.5-100.0) | 94.7% (74.0-99.9) | 87.8% (83.2-91.6) | 0.68 (0.61-0.75) |
| **R3** | **medication** | **61.2% (46.2-74.8)** | **99.6% (97.5-100.0)** | **96.8% (83.3-99.9)** | **92.2% (88.1-95.2)** | **0.80 (0.73-0.87)** |
| R4 |  | 24.5% (13.3-38.9) | 99.6% (97.5-100.0) | 92.3% (64.0-99.8) | 85.8% (81.0-89.8) | 0.62 (0.56-0.68) |
| R5 |  | 22.4% (11.8-36.6) | 99.6% (97.5-100.0) | 91.7% (61.5-99.8) | 85.5% (80.6-89.5) | 0.61 (0.55-0.67) |
| S1 | **Other combinations** | 67.3% (52.5-80.1) | 98.2% (95.5-99.5) | 89.2% (74.6-97.0) | 93.2% (89.3-96.1) | 0.83 (0.76-0.89) |
| **S2** |  | **75.5% (61.1-86.7)** | **98.7% (96.2-99.7)** | **92.5% (79.6-98.4)** | **94.9% (91.2-97.3)** | **0.87 (0.81-0.93)** |
| S3 |  | 10.2% (3.4-22.2) | 99.6% (97.5-100.0) | 83.3% (35.9-99.6) | 83.6% (78.6-87.8) | 0.55 (0.51-0.59) |
| S4 |  | 60.6% (42.1-77.1) | 99.2% (95.9-100.0) | 95.2% (76.2-99.9) | 91.0% (85.2-95.1) | 0.80 (0.71-0.88) |
| S5 |  | 75.8% (57.7-88.9) | 98.5% (94.7-99.8) | 92.6% (75.7-99.1) | 94.2% (89.0-97.5) | 0.87 (0.80-0.95) |
| S6 |  | 65.3% (50.4-78.3) | 98.7% (96.2-99.7) | 91.4% (76.9-98.2) | 92.9% (88.9-95.8) | 0.82 (0.75-0.89) |

*There were no events for these definitions, therefore sensitivity, specificity could not be calculated.

Shading represents definition categories. **Bold** text represents the best performing case definition per category.

CI, Confidence interval; PPV, positive predictive value; NPV, negative predictive value; ROC, receiver operating characteristic.
